# A Critique of the Stenger Test

**DOI:** 10.3390/audiolres15050115

**Published:** 2025-09-09

**Authors:** Andrew Bell, Myriam Westcott, W. Wiktor Jedrzejczak

**Affiliations:** 1 Eccles Institute of Neuroscience, John Curtin School of Medical Research, Australian National University, Canberra, ACT 2601, Australia; 2 DWM Audiology, Heidelberg, VIC 3084, Australia; myriam@dwmaudiology.com.au; 3 Institute of Physiology and Pathology of Hearing, ul. Mochnackiego 10, 02-042 Warsaw, Poland; w.jedrzejczak@ifps.org.pl; 4 World Hearing Center, Ul. Mokra 17, 05-830 Kajetany, Poland

**Keywords:** functional hearing loss, single-sided deafness, nonorganic hearing loss, pseudohypoacusis, middle ear muscles, central masking

## Abstract

Introduction: Most audiometers have an in-built “Stenger test” setting. The test is sometimes applied in cases of single-sided deafness as an indicator of malingering. Although textbooks have been written about it, the underlying conditions remain enigmatic. The literature usually points to psychological problems, pointing to the patient as having “nonorganic hearing loss”, “malingering”, “false and exaggerated hearing loss”, “hysterical hearing loss”, or “pseudohypoacusis”. These are all non-objective features without a sound scientific base, and the test tends to blame the patient for providing non-repeatable hearing thresholds. Methods: This opinion piece looks at the literature surrounding the Stenger test and the factors that might cause hearing threshold variability and concludes that the test has a subjective basis that makes it unscientific. In our opinion, we also think it is ethically questionable to blame the patient for malingering when there are non-repeatable findings. In order to make the test scientifically valid, we frame a testable hypothesis: that the Stenger effect could be due to unrecognised contraction of the middle ear muscles in response to stimulation of the contralateral (worse-hearing) ear. That is, we suppose that bilateral contraction impairs thresholds in both the good and poor ear, so the subject can no longer hear a tone in their good ear which they previously could when their audiogram was established monaurally. Thus, we make the case that the subject is not malingering—they genuinely cannot hear the test tones in either ear. Discussion and Conclusions: We believe it is incorrect to blame the patient when the problem may lie with incomplete understanding of how the auditory system functions bilaterally. The test needs to be objectively investigated and perhaps reinterpreted in terms of hearing sensitivity in one ear being reduced by sound levels in the contralateral ear. If this is not possible, we suggest it would be better if the Stenger test were abolished.

## 1. Introduction

The audiogram measures the overall sensitivity of the hearing system, taking into account the status of the middle ear (possible conductive losses) and of the cochlea (sensorineural effects). Over the short term, these factors are considered fixed, making the audiogram the foundation stone of audiology. How bewildering, then, if a patient’s hearing were to fluctuate from test to test or even over the course of a single test.

A fluctuating hearing threshold is beyond current understanding, and, as we will see, sometimes the reaction of an audiologist to a positive Stenger test is to blame the patient, accusing them—thinly or heavily veiled—of all sorts of failings. It is regrettable that many audiologists have accused clients of malingering, hysterical hearing loss, feigning, or false and exaggerated hearing loss (FEHL). Other terms have included nonorganic hearing loss, functional hearing loss, pseudohypoacusis, conversion hearing loss, psychogenic deafness, simulated hearing loss, and dissociative deafness [1]. The authors cited in [1] suggest that appropriate management might involve counselling or psychiatric intervention. In a textbook about the condition, it has been called “a psychosocial disorder” [2]. In this opinion piece, the term nonorganic hearing loss (NOHL) is used in order to align with the current literature, although a better term, we suggest, might be unstable hearing thresholds. In the older literature, the term pseudohypoacusis was commonly employed (literally, false low hearing), although we believe that all such terms are pejorative and should be discarded. Employing a euphemism does not eliminate the implied lack of honesty on behalf of the patient.

The present text—bringing together three different perspectives—examines why an audiologist, personally committed to the hearing health of their clients, might be so baffled by an audiogram that they suppose a moral failing of their subject. What is the reason why a patient would lie to someone who is just trying to do their job?

The question poses a professional challenge. The repercussions of a faulty diagnosis are considerable, even though the issue probably affects only a relatively small percentage of cases of single-sided deafness in which a Stenger test is used. Nevertheless, most audiometers today still have an in-built facility to conduct a Stenger test (e.g., https://www.amplivox.com/education/knowledge-hub/how-to-perform-a-stenger-test, accessed on 18 July 2025); https://www.interacoustics.com/audiometers/ac40/support/stenger-test, accessed on 18 July 2025).

The issue first came to prominence in the 1950s and 1960s, when war veterans were leaving the military and seeking compensation for hearing damage [3]. The authors of this 1965 work related how some veterans would exaggerate their hearing losses to receive larger compensation, so that the stereotype for the Stenger test became “an adult male faker” (F.N. Martin in Peck [2]). Indeed, given difficult financial circumstances, one can understand how that might happen and why the test results might look unusual. The problem that developed was that all cases of inconsistent hearing results were regarded similarly, so that by 2011, the typical picture of NOHL was “a child female nonfaker” exhibiting “false and exaggerated hearing loss” or FEHL (ibid.).

This opinion piece, written by a hearing scientist (AB), a practising audiologist (MW), and an experimentalist in daily contact with hearing-impaired individuals (WWJ), examines a wide range of alleged cases of NOHL and finds that the term is inappropriate, particularly for children, because it accuses mostly innocent patients of being insincere or worse. In one online guide to Stenger testing, a dialogue between an audiologist and a patient is depicted, with the thought balloon of the patient reading “I hear that in my bad ear. I can’t respond or the audiologist will know I can hear,” to which the audiologist, armed with his Stenger test, replies, “You’re caught” (https://issuu.com/adrianasmit; accessed on 18 November 2017).

Offering sympathy in a difficult situation, Zhao and colleagues recommend that “the child should not be confronted with the ‘lie’ and should be treated as though the hearing loss is real” [4] (p. 491). We wish to suggest that such statements are misguided. They are almost certainly false in cases where the subject is able to “almost phenomenally” register repeatable elevated thresholds, as if they had “an internal anchor” [5]. That anchor point, it is suggested here, may have a biological basis. It may be a threshold corresponding to an unappreciated sound sensitivity in the auditory system, perhaps even in the middle ear muscles (MEMs), and this paper sets out an argument derived from overlooked evidence from the literature that supports that position. It is our hope that by drawing attention to the inadequacy of the Stenger test, which is the prime test used to measure unilateral “non-organic hearing loss”, the whole suite of tests employed to detect NOHL may be brought into question and, if necessary, retired.

## 2. Background of the Stenger Test

The Stenger test was developed in Germany in the early 1900s [6] as a test for “simulation” and “dissimulation” of hearing in cases of unilateral deafness. It was originally based on tuning forks but later refined [7] until reaching the version installed in modern audiometers. Peck [2] describes a researcher in 1923 praising the Stenger test as “easy and infallible” for detecting simulated total hearing loss in one ear. However, as Altshuler [7] relates, the test has attracted confusion, and this overview suggests it should be abandoned or at least totally reframed.

Nowadays, cases of unilateral hearing loss are readily resolved by offering the patient a hearing aid or cochlear implant, so that circumstances for simulation or malingering have been greatly reduced. Nevertheless, the Stenger test continues to be built in to modern audiometers and is said to be useful in medico-legal cases seeking compensation for occupational hearing loss [1,8].

The idea behind the Stenger test is that if two identical tones are separately but simultaneously presented to each ear—either over tuning forks or headphones—the subject will only be aware of the sound in the ear with greater loudness. However, in cases of single-sided deafness (SSD) where there is a 40 dB or more difference in threshold between the ears, a curious phenomenon, the Stenger effect, can occur. Even though a tone is applied to the good ear above threshold, the subject will say they can no longer hear it when another tone, below threshold, is presented to the poorer ear. The non-reporting of a tone that should be audible was interpreted by Stenger as proof of malingering [9] and has been used as a clear demonstration of what Peck prefers to call “false or exaggerated hearing loss” (FEHL) [2,10]. In chapter 2 of his book, Peck describes the case of positive Stenger as one in which the patient “hears the signal in the worse ear” but is “unwilling to react”. Scientifically, this is unwarranted speculation, as if the experimenter knows more about what is in the patient’s head than the patient does. Elsewhere, Peck speaks of a “false portion” of the audiogram; another where there was “an exaggeration by 40-dB”; and one where a person “wishes to present a hearing loss [so] does not respond.”

Martin [8] portrays the Stenger test as “an efficient test for quick identification of unilateral non-organic hearing loss.” The only equipment required is a pure tone audiometer with separate level settings for the right and left ears. Sometimes a Bekesy audiometer is used, and at other times speech samples are.

**Figure 1:**
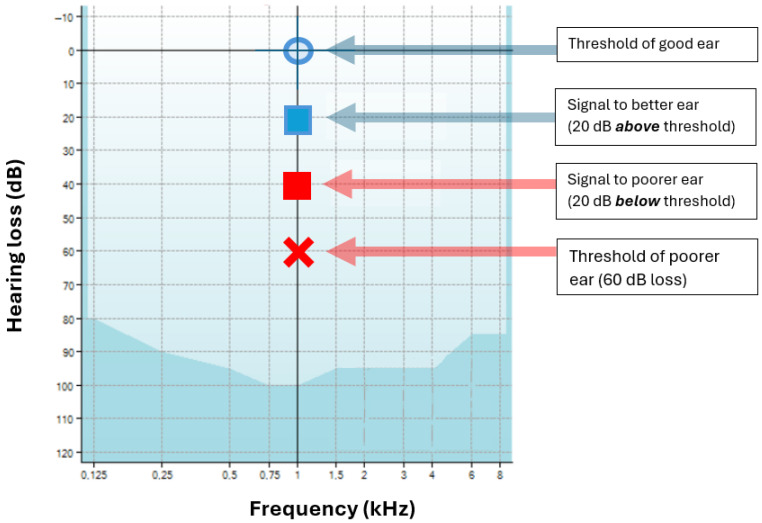
Illustration of the Stenger effect. In this hypothetical case, the subject has a monaural threshold of 0 dB in their good ear (blue circle) and 60 dB in their poorer ear (red cross). A signal of 20 dB is applied to their good ear (blue square), which is audible. But when a signal of 40 dB is applied to their poor ear (red square), which is below its threshold, a subject will sometimes report they now hear nothing at all—the tone in the good ear is no longer audible (positive Stenger). The patient may now be accused of lying. Image adapted from “Stenger test”, Wikipedia (CC-BY-ND 4.0).

The Stenger effect is illustrated in Figure 1 for a case of SSD in which the poorer ear has a threshold of 60 dB HL at 1 kHz while the good ear has a threshold of 0 dB HL. The figure illustrates that when a tone of 20 dB is applied to the good ear (which should be audible) at the same time as a tone of 40 dB is applied to the poorer ear (which should be inaudible), the subject claims that nothing is audible. Is this really true, or is the subject “malingering”? Some audiologists think that the subject is malingering (a ‘positive’ Stenger), while others have objected and said that the audiologist should always respect their patient’s integrity. Complicating matters, many cases of positive Stenger have come from children, who, lacking full autonomy, can be more easily persuaded to conform with what their elders want [2,4,11].

One attractive feature of the Stenger test is that it can sometimes deliver a numerical measure of how much (in dB) the subject is “exaggerating” their loss. Thus, by adjusting the intensity in the poor ear until the point of “no response” is reached, the “minimum contralateral interference level” (MCIL) can be measured [2] (ch. 7). The MCIL can be surprisingly constant [2] (ch. 2). Peck says that, in certain cases, the Stenger effect can be demonstrated in ears with threshold differences of only 20 dB [2].

## 3. The Stenger Test in Practice

**Figure 2:**
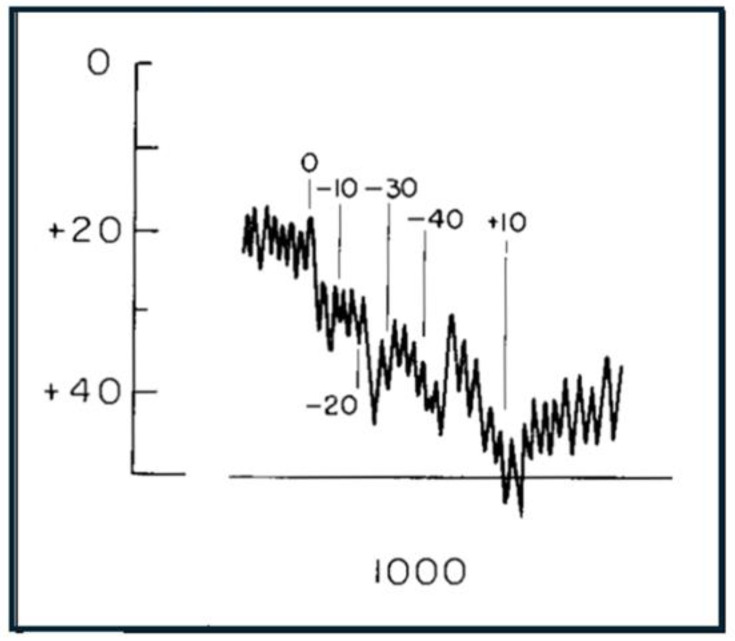
An example from the literature of “functional loss” in a subject with single-sided deafness of >40 dB in the left ear (from Watson and Voots [12]). Bekesy traces show perceived threshold of a 1000 Hz tone applied to both ears when levels in the deaf ear were steadily increased (attenuator settings switched in 10 dB steps as marked). The traces indicate that thresholds become more impaired as the tone applied to the poorer ear is made louder. On this basis, Watson and Voots diagnosed this “contradictory” behaviour as indicating functional hearing loss—the subject was indicating they could not hear the tone even though the tone “must have been” constantly audible in the good ear. Reproduced with permission of Taylor & Francis (Oxfordshire, UK) through CCC Ltd.

Although there have been many words, and much speculation, there is little actual published data on the Stenger test. This perhaps raises a warning flag. One example of NOHL using a modified form of the Stenger test appears in Watson and Voots [12] and is shown in Figure 2.

A notable feature of the book by Peck [2] on what he calls “pseudohypacusis” (false low hearing) is that no actual examples are provided, although there is a table of “hypothetical” cases (his Table 7-1). There is a column headed “overlay”, which indicates “how much of the loss is false”, and here, the overlay in positive Stenger cases is assumed to range from 35 to 50 dB. Peck’s table is designed to illustrate what happens in cases of malingering. In a later section, we introduce another possible explanation involving bilateral action of the middle ear muscles, a possibility that suggests that malingering may be confounded with a real physiological effect, one that is recorded numerically as MCIL. This contribution asks, what is the actual origin of the MCIL? Some possibilities are that the effect might be due to the middle ear muscles, bone conduction, or a central masking effect (e.g., [13,14]).

## 4. Critiques

The Stenger test is surrounded by a long history of confusion and controversy [2] (ch. 7); [7]. After all, it is strange that a patient provided correct indications of thresholds when the monaural audiogram was measured but then began to lie when a not dissimilar test (the Stenger test) was conducted. A contributing factor may be that there might be an air of suspicion in the clinic, and, indeed, the problem of false and exaggerated hearing loss first came into prominence after World War II when many soldiers were returning to civilian life with disorders associated with military conflict and were seeking compensation [2]. The tenseness of the medico-legal situation may have meant that cooperative feelings in the clinic were absent, so that the traditional case became that of an “adult male faker” (Foreword to Peck [2] by F.N. Martin, who categorised the condition as a psychosocial disorder). Peck (ch. 2) also alludes to a general rise in compensation cases as a prime cause of what he calls FEHL and relates unfortunate cases where the U.S. Army resorted to the use of sodium pentothal (“truth serum”) and heavy use of suggestibility when false hearing loss could not be resolved through other means, including hypnosis.

The psychology of the situation is potentially upsetting. According to Martin [8], there can sometimes be an unfortunate stigma surrounding the Stenger test. Martin says that once individuals have been diagnosed as malingering or uncooperative, their reputations and prestige may be damaged. “To label a patient in such ways is a grave matter,” he says, “since it implies deliberate falsification. Such labels are difficult to expunge and may be tragically unjust.”

According to Noble [15], a turning point was the publication in 1965 of a collection of papers edited by Ventry and Chaiklin [3]. Noble describes the work as transforming the “immorality” of malingering into the “illness” of functional hearing loss (p. 1). The collection paints a disjointed picture, where we learn that, of 45 subjects, “20 subjects continued to present functional hearing loss despite repeated counselling and testing” (paper 6) and “persistence of functional hearing loss … [may relate] … to lower intelligence … excessive moral virtue, and a tendency toward indirect rather than direct means of expressing hostility.”

However, Noble is clear in saying that, no matter what the label, any assessment system that is itself fake can only give rise to faking. He thinks that anyone who is faking on a test cannot be described as having functional hearing loss, and that the term is an empty one and should be discarded. Nearly 40 years later, the remarkable aspect is that the Stenger test is still being offered as an audiological tool, and textbooks still contain expositions of how to use it. *The Professional Practice Guide* (2022) of Audiology Australia refers to the Stenger test in connection with sub-domain 4.8 (functional/non-organic hearing loss assessment), and in 2019 the American Academy of Audiology discussed the appropriate terminology to use for fake and exaggerated hearing loss [10]. According to Peck, the term FEHL identifies the key aspect of what is involved—its falseness.

According to Martin [8], a positive Stenger test “proves” that the indicated threshold is incorrect. However, later discussion reveals that the accuracy and efficiency of the Stenger test are widely contested. Thus, in one group of 225 patients with non-organic hearing loss, the Stenger test was applicable to only 57% of them and it had the poorest efficiency of all the tests tried, correctly labelling only half.

According to the history of the Stenger test by Altshuler [7], confusion surrounding the test began soon after various modifications and improvements were made in the middle of the last century, and opinions started to diverge. Although many researchers thought the test was worthwhile, negative opinions began to accumulate, and Altschuler quotes one investigator (Hood) as saying that “the procedure is seldom of value.” There seemed to be some agreement in the 1950s and 60s that the test was only really useful for monaural cases of malingering, but for bilateral cases the level of confusion mounted, and the ready availability of hearing aids rendered the test less useful.

Over time, the stereotype for functional hearing loss became “a child female nonfaker” (foreword by Martin to Peck [2]). An interesting profile of the “pseudohypacusic” was earlier given by Martin [8], who used the term for anyone whose auditory threshold was inflated above the patient’s “true” organic threshold as the result of conscious or unconscious motivation.

Although himself a proponent of the Stenger test, Peck [2] devotes a section of his chapter 7 to a wide-ranging critique of it. He finds that the success of the test varies widely, with some reports claiming that it detects 42% or 48% of FEHL cases and others claiming 100% effectiveness. The confounding factor in all this, however, is that many of the supposed tests of performance explicitly ask normal-hearing volunteers to “simulate” having hearing loss in one ear while the investigator tries to catch them out. This adds one strange methodological twist upon another and makes it hard to assess the results. Were the original subjects actually simulating in the same way, or were they suffering from a physiological condition perhaps affecting their middle ear muscles? Peck perhaps approaches the core of the matter when he reports one researcher (Ventry) cautioning against generalising from normal-hearing simulators to real patients with FEHL, as the differences may outweigh the similarities. Indeed, our view is that the whole issue has become a confused mixture of overlooked physiological and psychological factors compounded by needless argument. Actual bilateral effects need to be experimentally explored.

We do not deny that sometimes there are cases where patients are either confused or wish to fail a hearing test, but such cases need to be treated by a psychologist, not an audiologist, and are best kept out of the audiological literature.

## 5. An Audiologist’s Perspective

As an audiologist who specialises in the assessment and rehabilitative care of patients with a complex audiological history, I follow a biopsychosocial, patient-centred approach, working within a collaborative multidisciplinary team. This is not only because of better outcomes for my patients but because this aligns with my ethical and moral approach to clinical practice.

Pure-tone audiometry is a subjective, behavioural measurement of a hearing threshold, relying on patient responses to faint stimuli heard in a sound-proof booth using calibrated equipment. This requires cooperation, alertness, and concentration from both patient and audiologist. Some degree of test–retest variability is commonplace between clinics and even in the same clinic and is typically ±5–10 dB.

An experienced audiologist will notice inconsistencies in responses or discrepancies between the results and the patient’s clinical presentation, history, and communication ability. To ensure better accuracy, stimuli can be presented randomly, at nonpredictable intensities, or only at low intensities [16].

Objective measures, like otoacoustic emissions (OAEs), auditory brainstem responses (ABRs), cortical auditory evoked potentials (CAEPs), and acoustic reflex thresholds can be used to distinguish between organic and non-organic hearing loss. The Stenger test is a behavioural test, based on the premise of catching out a person who is knowingly exaggerating, feigning, or malingering a hearing asymmetry.

Austen and Lynch (2004) proposed a model of NOHL to bring a more nuanced view to the distinction between (a) NOHL that is either consciously feigned and does not warrant psychological intervention and (b) NOHL that is unconscious, is serving a psychological purpose, and requires psychological or psychiatric intervention [17]. The authors proposed three categories of NOHL on a continuum: malingering, factitious, and conversion disorder. Diagnosis depends on motivation, which is assessed by the degree of intention and nature of the gains. Individuals might move between the categories, and management strategies might differ too. This model has been influential and widely adopted [16].

I regularly, but not frequently, see patients with a complex audiological and psychological history, such as acoustic shock disorder or post motor vehicle accident SSHL, who provide hearing thresholds that are consistent and repeatable but are inconsistent with their clinical presentation, history, and communication ability. A proportion of these patients fit a likely diagnosis of involuntary conversion disorder/functional hearing loss, which is not necessarily fixed and can be situation-specific (pers. comm.).

Each NOHL patient needs to be carefully and respectfully considered from a biopsychosocial perspective. While the Austen and Lynch model provides a considered perspective, it has, as the authors themselves point out, retained pejorative vocabulary inconsistent with a person-centred approach [16,17]. Some patients may well respond inaccurately with intent, and it is reasonable in this context for an audiologist to provide a non-judgmental comment. All forms of NOHL, in particular a suspected diagnosis of conversion disorder/functional hearing loss, require an objective audiological test to confirm them. Where this sits on the Austen–Lynch continuum should be a joint diagnosis, made in a multidisciplinary setting by a psychiatrist, psychologist, neurologist, and ENT specialist. It is not the role of the audiologist to assess the likely financial or emotional gains of the patient.

The Stenger test, based on catching out a patient, contravenes a person-centred and biopsychosocial approach and reflects a paternalistic, patronising, and outdated approach to patient care. Additionally, it is intended to be a confrontational test, with the potential to lead to psychological harm. It is time it was dropped.

—MW

## 6. An Experimental Scientist’s View

In modern practice, the Stenger test can be replaced by objective alternatives that minimise subjective interpretation, rely on measurable physiological responses, respect patient dignity, and are supported by empirical scientific evidence. Objective approaches include otoacoustic emissions (OAEs), auditory brainstem responses (ABRs), auditory steady-state responses (ASSRs), cortical auditory evoked potentials (CAEPs), immittance audiometry (including acoustic reflex testing), and electrocochleography (ECochG). They not only yield more reliable, physiologically grounded results but also uphold patient dignity and ensure accurate diagnoses in suspected SSD and malingering cases.

OAEs offer a quick, non-invasive way to measure cochlear function by assessing the detection of sounds by outer hair cells in response to auditory stimulation [18]. OAEs require minimal patient cooperation and help confirm cochlear integrity when SSD is suspected [19], although they do not address higher-level auditory processing.

ABR tests measure electrical activity in the auditory nerve and brainstem via scalp electrodes, providing frequency-specific hearing thresholds and detailed insights into the auditory pathway. ABRs remains unaffected by patient motivation, making them particularly useful for verifying asymmetrical hearing loss in potentially non-cooperative individuals [20]. ABRs can also be replaced with ASSRs [21], which provide similar information but can be measured in a fully automated manner, albeit with some trade-off in repeatability.

CAEPs, on the other hand, capture neural activity in the auditory cortex and thus reflect conscious auditory perception [22]; their resistance to conscious suppression can be instrumental in identifying malingering, although they can be more time-consuming, costly, and reliant on patient relaxation.

Immittance audiometry, which includes tympanometry and acoustic reflex measurements, reveals critical information about middle ear function and muscle activity [23]. While it does not provide direct measures of hearing sensitivity, an intact acoustic reflex pathway can challenge claims of profound hearing loss by confirming functional integrity of the stapedius reflex.

ECochG further refines diagnosis by capturing electrical potentials from the cochlea and auditory nerve, offering greater detail than OAEs and enabling differentiation between sensory and neural components of hearing loss. Although more invasive than other tests, it helps clarify complex pathologies [24].

Despite the recognised weaknesses and ethical questions surrounding the Stenger test, it remains in circulation because of its speed and simplicity, whereas more objective methods often demand specialised equipment, training, and time. However, recent developments such as sweep OAE protocols [25], advanced ECochG techniques [26], and smartphone-based hearing assessments [27] show that more efficient and affordable objective measures are available. As these technologies evolve and their costs continue to decrease, streamlined and automated testing procedures hold the potential to render the Stenger test obsolete, thereby eliminating the need for a method built on questionable scientific and ethical foundations.

—WWJ

## 7. A Neurophysiological Explanation

After reviewing the literature surrounding the Stenger test, one of us (AB) believes a neurophysiological explanation for the phenomenon is worth exploring. The hypothesis is scientifically testable and avoids blaming the patient. It is suggested that the Stenger effect could be due to bilateral contraction of the middle ear muscles, a reaction promoted by the elevated sound levels applied to the poorer ear (≥40 dB SPL). Although such levels are below normal reflex threshold (typically 85 dB SPL), they are sufficient to initiate slow contraction of the muscles.

This hypothesis incorporates three major factors.

**Figure 3:**
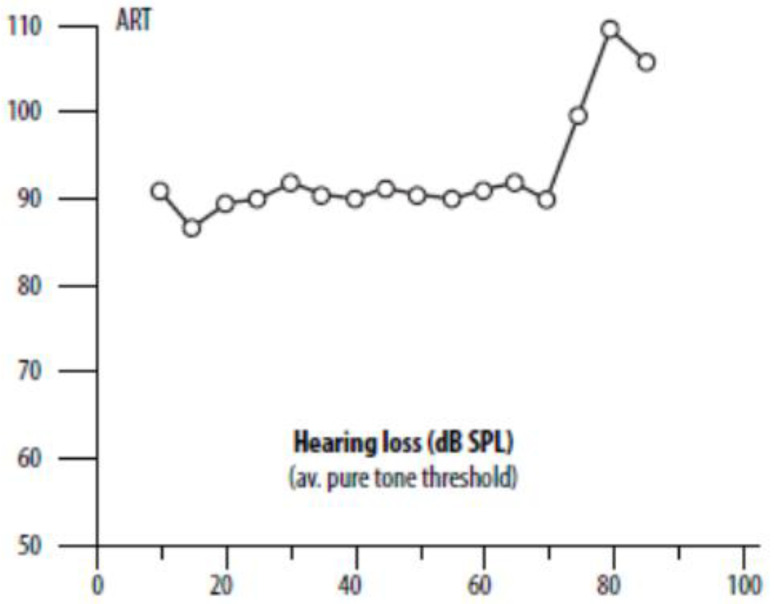
Remarkably, the threshold for reflex contraction of the middle ear muscles is independent of hearing loss, at least up to 70 dB SPL, as indicated here. The *x*-axis is the average amount of hearing loss in dB SPL, and the *y*-axis is the acoustic reflex threshold (ART, dB SPL) as measured in the contralateral ear and elicited by a pure tone in the ipsilateral ear. A logical inference is that the reflex is being triggered not via the cochlea but by the middle ear muscles themselves, whose vibration sensitivity will be independent of the inner ear. The plot is from Hyde et al. [28] based on 1207 subjects. The hearing loss is the average of thresholds at 0.5, 1, 2, and 4 kHz. Used with the permission of Taylor & Francis through CCC.

The first is the largely unappreciated factor that the threshold for contraction of the middle ear muscles is independent of hearing loss [28,29,30]. This nonintuitive fact is illustrated in Figure 3 and supports the idea that a person with SSD is likely to be particularly susceptible to a positive Stenger. Referring again to Figure 1, the Stenger test involves applying a tone some 10–20 dB weaker than a subject with a unilateral hearing loss can hear, so a person with a loss of 60 dB in one ear might receive a tone of 40 dB, which is within 40 dB of the nominal reflex threshold.

A second major factor is that the middle ear muscles are dynamic and are constantly adjusting their tension [31,32]. This means there is likely to be some degree of contraction even at levels considerably below the reflex threshold. In brief, some effects can be seen tympanometrically as low as 20–30 dB SPL, while reflex effects on OAEs can also be seen at low SPLs. Thus, Kawase and colleagues [33] found that contralateral noise at a level of only 20–30 dB SPL can, in normal listeners, cause a 2–3 dB elevation in auditory threshold at mid frequencies, with the effect increasing with noise level. Boothalingam and Goodman [34] found that MEMR thresholds as measured by OAEs were about 20 dB lower than those measured with a conventional tympanometer, and similar results were reported by Feeney and colleagues [35].

Thirdly, the middle ear muscles of some subjects are more sensitive than normal and can show contraction tens of decibels lower than expected. In some cases, reflex effects can be seen as low as 40 dB SPL [36,37].

Taken together, one can appreciate that a proportion of individuals with SSD might have particularly sensitive middle ear muscles which could physiologically react at sound levels well below the normal reflex threshold. By extension, it is even possible that in these individuals, the middle ear muscles might be more sensitive to sound (actually vibration) than the cochlea itself (although such an idea was deemed impossible by Jerger and colleagues [38]).

If the middle ear muscles contract, then sound transmission through the middle ear will be attenuated. The muscles act as the cochlea’s gate-keepers, and their role is to quickly protect the cochlea from loud, damaging sound [31]. Even if a sound is below full reflex threshold, there will be some attenuation, and the ear will show reduced sensitivity. The middle ear reflex is bilateral, so during a Stenger test, the good ear will also be affected. In that case, a sound that may be only 10–20 dB above the hearing threshold might become inaudible. The attenuation provided by the middle ear muscles is not easy to measure, but there is some evidence that the attenuation may reach as much as 20 dB at 1 kHz (see Figure 3 of [31], which amalgamates the findings of [39,40]). More recent work has shown smaller effects, some as little as 1 or 2 dB [41,42,43,44], but logically, one might ask why a system as anatomically sophisticated as the middle ear muscles should, in practice, produce such small effects.

Feldman [45] considered the possibility that the acoustic reflex might underlie cases of pseudohypoacusis, but he dismissed the idea that an inaudible tone could stimulate the reflex. The point to consider, however, is that the sound does not have to be at a level that induces full reflex activity, only sufficient to induce a degree of contraction. Clearly, more experimental work on low-level middle ear muscle contraction is needed. The speculations made here are intended to prompt such efforts.

Martin [8] is entirely unconvinced that the middle ear muscles may be involved. He suspects experimental artefacts, saying that “the suggestion that the acoustic reflex may be achieved by a tone which cannot be heard must be rejected, and a diagnosis of pseudohypacusis may be made” (p. 757). Admittedly, responses to inaudible sounds appear counterintuitive, although such an unconventional possibility has been considered, e.g., in [30], where it is argued that there may be a sensing mechanism associated with vibration of the eardrum, to which the middle ear muscles are intimately connected. It is pointed out that all muscles are exquisitely sensitive to vibration, so that ultimately experiments are needed to settle the question. It is possible that the Stenger effect may actually be part of a larger puzzle.

To be clear, it is not being suggested that it is the spasmodic acoustic reflex threshold that is abnormally sensitive; instead, it is suggested that there may be a lower threshold at which steady contraction of the middle ear muscles progressively begins. This could be at a level below the reflex threshold and will naturally vary from subject to subject.

In some cases, MEM contraction might be due to contraction of the tensor tympani alone, which is known to fluctuate due to environmental and emotional conditions, including touch to the face and ears and even by headphones [31,46,47,48,49]. (See also [50,51], which looks at objective measures of temporary threshold shift in hyperacusis patients). There is evidence the tensor tympani can contract in response to sound levels below those known to trigger the middle ear reflex [47]. In other words, the hearing system may in certain respects be more sensitive than our current measuring instruments. Moreover, some individuals have “negative middle ear pressures”, which can persist for months, and Bell [52] has interpreted this as a long-term, partial contraction of the tensor tympani. In terms of tympanometry, this can be interpreted as contraction of the tensor tympani masquerading as a type C tympanogram [52]. An alternative explanation is that the Stenger phenomenon may be due to partial contraction of the stapedius (the muscle responsible for the acoustic reflex) at sub-reflex levels [31].

There are certain other anomalies surrounding the Stenger test, and these are worth drawing attention to.

Firstly, Peck [2] notes that many false responders can be quite consistent during testing (his Ch 1). Indeed, some individuals have “a phenomenal ability” to repeat their supposedly made-up thresholds [5] (p. 64). Similarly, Hooper [53] noted “surprisingly consistent” audiometric responses in a subject complaining of acoustic shock and was even able to construct an audiogram (his Figure 2), although the Stenger effect was only evident at three specific frequencies.

Secondly, it is also worth pondering the logic of the question asked by Gelfand and Silman [54] when they studied NOHL: why might a patient with SSD ‘choose’ to exaggerate their loss in one ear only? Further, to repeat, why should a subject provide honest audiograms monaurally but then begin to lie when a bilateral Stenger test is conducted? A useful survey of what is called functional hearing loss and its puzzling anomalies can be found in Baguley et al. [16], where it is pointed out how, historically, the audiology literature has equated functional hearing loss with malingering. This confusion needs to be sorted out, and more research is needed.

Ward [13] found that, in normal subjects, there was an appreciable reduction in hearing sensitivity due to sound in the contralateral ear, but this effect was seen only at high sound pressure levels. Ward could not separate the effects of reflex activation and central masking. In subjects with single-sided deafness, the situation is more complex, and again further investigation is called for.

Finally, it is noteworthy that Martin [8] (p. 762) records that admissions of lying are exceedingly rare and cautions that value judgments are not within the purview of the audiologist.

Taken together, all these ideas confirm that something strange is going on with the Stenger test. At the very least, acknowledgement is needed that obtaining consistent results is impossible if the client is actually lying. Here, Gelfand’s idea of internal ‘anchor points’ based on reflex thresholds is an appealing notion, and this too calls for further investigation.

—AB

## 8. Towards Integration

This opinion piece has endeavoured to show how the Stenger test is out of step with scientifically sound practice. It needs to be either abandoned or objectively investigated in order to more fully understand the effects of bilateral stimulation in cases of SSD. It is perhaps possible that the Stenger test might be explained as an instance of partial, bilateral contraction of the middle ear muscles. Although some may consider this unlikely, it does provide a possible explanation. In such a case, diminished thresholds could occur in the better ear, with the audiologist unwittingly blaming the subject for deviating from the previously established (monaural) audiogram. It is advisable for audiologists to always keep an open mind, guided perhaps by the statement from Sherlock Holmes that whenever the impossible has been eliminated, whatever remains, however improbable, is the truth.

Audiologists always need to be on the side of the patient and should not question their client’s integrity by using loaded terms like nonorganic hearing loss or related euphemisms. We suggest such terms should be withdrawn because they refer to a flawed technique based on invalid assumptions. We think the Stenger test does not align with science-based audiology and should be retired. Perhaps a more neutral term for bilaterally induced threshold shifts is “unstable hearing thresholds”.

There are documented cases where hearing levels have been observed to fluctuate depending on middle ear muscle tension—due, for example, to touch to the face or ears [50]. More exploratory work along the lines of [51] is needed. It is possible that certain individuals might be prime candidates for unstable hearing thresholds, and in this context, hyperacusis and acoustic shock are relevant [55,56].

Westcott advocates “person-centered care”, a process respecting a person’s preferences and values. It is the foundation of high-quality healthcare and has been shown to improve patient outcomes. The World Health Organization defines people-centred care as extending the concept of patient-centred care to individuals, families, communities, and society (https://iris.who.int/handle/10665/252698, accessed 18 July 2025).

Shared decision making, where clinicians and patients work together, is based on clinical evidence and the patient’s informed preferences and is now the legally accepted standard in the UK [57]. Courts now take the view that patients should be helped to make informed choices, so simply providing information about an investigation or treatment chosen by a clinician and obtaining a signature on a consent form is no longer sufficient.

In Australia, the Commission on Safety and Quality in Health Care promotes person-centred care within all healthcare organisations. Their Charter of Healthcare Rights says a patient needs to be treated with dignity and respect; further, before informed consent can be given, they should be provided with clear information about their condition and the possible benefits and risks of various tests and treatments (https://www.safetyandquality.gov.au/our-work/partnering-consumers/australian-charter-healthcare-rights, accessed 18 July 2025).

To fully understand a person’s medical condition, Engel proposed a ‘biopsychosocial model’ in which, alongside biological factors, psychological and social factors need to be considered [58]. A biopsychosocial model has a reasonable claim to be the overarching framework for medicine and healthcare and is now used in clinical and health educational settings worldwide [59]. Again, a multidisciplinary approach is essential.

## 9. Limitations

A limitation of this text is that it sets out an opinion and does not include original experimental data. It would be of interest to test the middle ear muscle hypothesis, although we acknowledge it would require considerable resources. At the same time, it would be difficult to actually find a group of subjects, let alone experimenters, who would be willing to undertake a direct comparison with the Stenger test as originally framed. Finding “data” for a Stenger test is indeed hard, and perhaps this is one reason why it has persisted so long without being successfully challenged.

Moreover, while we recommend objective tests, some (such as cortical auditory evoked potentials, CAEPs), still call for a good level of patient cooperation and involve considerable complexity. Be that as it may, such a test provides a better alternative than a subjectively based Stenger test.

## 10. Conclusions

This text challenges a long-standing practice in audiology, urging a shift toward more ethical and scientifically grounded methods. We call for further research to validate alternative hypotheses and to refine diagnostic tools for single-sided deafness where “non-organic hearing loss” appears most prevalent. Our conclusion is that the Stenger test is an unreliable tool and should be dispensed with. In most cases involving the Stenger test, it is likely that the blame lies with a failure to fully understand how the auditory system functions in cases of SSD.

Of course, we do not deny that occasional cases of feigned hearing loss exist, but it is wrong for audiologists to make that accusation when the changes in thresholds may have a biological or involuntary basis.

What is the appropriate reaction on learning that an honourable profession may have gone astray for more than a century? On the positive side, it appears as if the use of the Stenger test is fading, but traces of it remain. It is unfortunate that no association of audiologists appears to have ever admitted that mistakes were made or has apologised to those who may have suffered harm.

We believe it is in the interests of the profession to make a clear admission that mistakes have been made and that no more cases of patient-blaming will occur. As Noble [15] says, if there is any malingering going on, that is not for the audiologist to judge but rather a case for lawyers and judges.

Looking back, however, the Stenger story appears to be a cautionary tale of how a scientifically based profession, claiming to be based on verifiable facts, seems to have deviated from its empirical foundations and proceeded in an unhelpful and even harmful direction. Our view is that the history of the Stenger phenomenon reveals some regrettable aspects that need to be quickly rectified.

## Data Availability

No new data were created or analysed in this study. Data sharing is not applicable to this article.

## Abbreviations

The following abbreviations are used in this manuscript:

FEHL	False and exaggerated hearing loss
dB HL	Decibel hearing level
kHz	Kilohertz
MEM	Middle ear muscle
SPL	Sound pressure level
ECochG	Electrocochleography
OAEs	Otoacoustic emissions
ABR	Auditory brainstem response
MCIL	Minimum contralateral interference level
